# Indications for Isolated Arthroscopic Bankart Repair in the Adolescent Shoulder May Be Limited: Bipolar Bone Loss and Low Distance to Dislocation Found Commonly in an Adolescent Population With Glenohumeral Instability

**DOI:** 10.1177/03635465261483327

**Published:** 2026-09-22

**Authors:** John E. Arvesen, Henry B. Ellis, Dang-Huy Do, Cody Todesco, Antonia L. Dinulescu, Robert L. Van Pelt, Charles W. Wyatt, Philip L. Wilson

**Affiliations:** † Scottish Rite for Children/UT Southwestern Medical Center, Dallas, Texas, USA; Investigation performed at Scottish Rite for Children, Dallas, Texas, USA

**Keywords:** adolescent shoulder instability, bipolar bone loss, distance to dislocation, Hill-Sachs lesion, glenoid bone loss, Bankart repair, remplissage

## Abstract

**Background:**

Bone loss is an established risk factor for recurrent anterior shoulder instability and failure of isolated arthroscopic Bankart repair; however, its prevalence and clinical significance at initial presentation in adolescents remains poorly characterized.

**Purpose/Hypothesis:**

The purpose of this study was to evaluate the prevalence of bone loss and the factors that correlate with bipolar bone loss in adolescent patients evaluated with anterior shoulder instability. It was hypothesized that male sex, older age, higher number of dislocations, and contact sports participation would be associated with bipolar bone loss at initial presentation.

**Study Design:**

Cohort study; Level of evidence, 3.

**Methods:**

A retrospective institutional review board–approved study of 181 patients aged 10 to 19 years diagnosed with anterior shoulder instability from January 2022 to March 2024 was performed at a single tertiary referral center. Glenoid bone loss, Hill-Sachs interval, on-track versus off-track status, and distance to dislocation (DTD) were measured on magnetic resonance imaging. The association between bipolar bone loss and patient characteristics, sports participation, and prior dislocation history was evaluated using chi-square and Mann-Whitney *U* tests with univariate and multivariate logistic regression.

**Results:**

Overall, 74.6% of patients exhibited bone loss; 34.3% had bipolar bone loss, 7.2% were off-track, and 41.4% had a DTD <10 mm. Among those with first-time dislocation, 69.2% presented with bone loss and 37.4% had a DTD <10 mm. Bipolar bone loss was significantly associated with older age (16.29 vs 15.61 years; *P* = .012), male sex (96.8% vs 69.7%; *P* < .001), high-impact/collision sports participation (94.6% vs 73.6%; *P* = .001), and ≥5 prior dislocations (28.8% vs 9.9%; *P* = .003). On multivariate analysis, male sex (OR, 14.20; *P* = .011) and ≥5 prior dislocations (OR, 3.22; *P* = .026) were independent predictors of bipolar bone loss.

**Conclusion:**

One-third of adolescents in our series presenting with anterior shoulder instability had bipolar bone loss at the initial evaluation, with male sex and ≥5 prior dislocations as independent predictors. The high prevalence of off-track lesions and DTD <10 mm suggests that isolated Bankart repair may be insufficient for many adolescent patients.

Anterior shoulder instability in adolescence is associated with a high recurrence rate and often results in increasing glenoid- and humeral-sided bone loss with each dislocation.^13,17^ Approximately 20% of all anterior shoulder instability occurs in patients <20 years of age, with a resultant 93% recurrence rate in the 5 years after nonoperative management.^10,19^ Factors correlated with recurrent instability include participation in contact/collision sports, male sex, age, arm dominance, and underlying hypermobility. Additionally, recent literature has demonstrated that combined humeral- and glenoid-sided bone loss may lead to an increased risk of recurrence.

Recent published series have documented the importance of addressing any existent bipolar bone loss to reduce the risk of recurrence.^12,30^ Historically, research has focused on glenoid bone loss as well as the on-track/off-track calculation to determine the best mode of treatment for patients evaluated with traumatic anterior shoulder instability.^2,9,14,21^ The influence of the Hill-Sachs lesion as a negative effect on outcomes of anterior shoulder stabilization has been increasingly noted. The literature provides little information about the incidence of Hill-Sachs lesions and distance to dislocation (DTD) measurements at the time of initial evaluation in adolescent patients with anterior shoulder instability.

Recognition of the additive effect of glenoid bone loss and a humeral Hill-Sachs lesion can influence surgical management of these injuries. The DTD calculation is a useful measurement to quantify the risk of this additive bone loss, and a DTD <10 mm has been cited as a risk factor for failure of treatment with isolated arthroscopic Bankart repair.^2^ The purpose of this study was to evaluate the prevalence and factors correlated with bipolar bone loss in adolescents with traumatic anterior shoulder instability. We hypothesized that male sex, older age, higher number of dislocations, and contact sports participation would be associated with increased bone loss at initial evaluation in adolescent patients. Also, more combined bone loss and a high frequency of DTD <10 mm would be noted within this population.

## Methods

This study was approved by our institutional review board with a waiver of informed consent.

### Study Patients

This is a retrospective review of patients aged 10 to 19 years diagnosed with traumatic anterior shoulder instability from January 2022 to March 2024 at a single institution. The electronic medical record system was utilized to identify all nonoperative and operatively treated patients with a diagnosis of traumatic anterior shoulder instability and who were evaluated with advanced imaging (International Classification of Diseases, 10th Revision codes S43.0-, S43.3, M24.41-, and M25.3-; and Current Procedural Terminology codes 29806, 23455, 23460, 23465, 23462, and 23466). There were 230 patients with advanced imaging who were treated for anterior shoulder instability. Exclusion criteria included history of seizure or syndromic disorder (n = 28), major concomitant injury (n = 10), and incomplete clinical data (n = 11), leaving 181 patients available for study.

### Data Collection

Patient characteristics, mechanism of injury, sports participation, and number of dislocations were collected through electronic medical records. Sports participation type was further categorized as low impact or high impact (Figure 1). Non–sport-related injuries were traumatic instability events such as a fall, Jet Ski injury, or roughhousing. The number of dislocations was categorized as first-time dislocation, 2 to 4 dislocations, or ≥5 dislocations. The variable represents total instability events, inclusive of both subluxations and dislocations, and includes the presenting event. The data set records first lifetime ipsilateral dislocation and prior instability events categorized as 0 or 1, 2 to 4, and ≥5; subluxation and dislocation were not routinely distinguished in charts.

**Figure 1.**
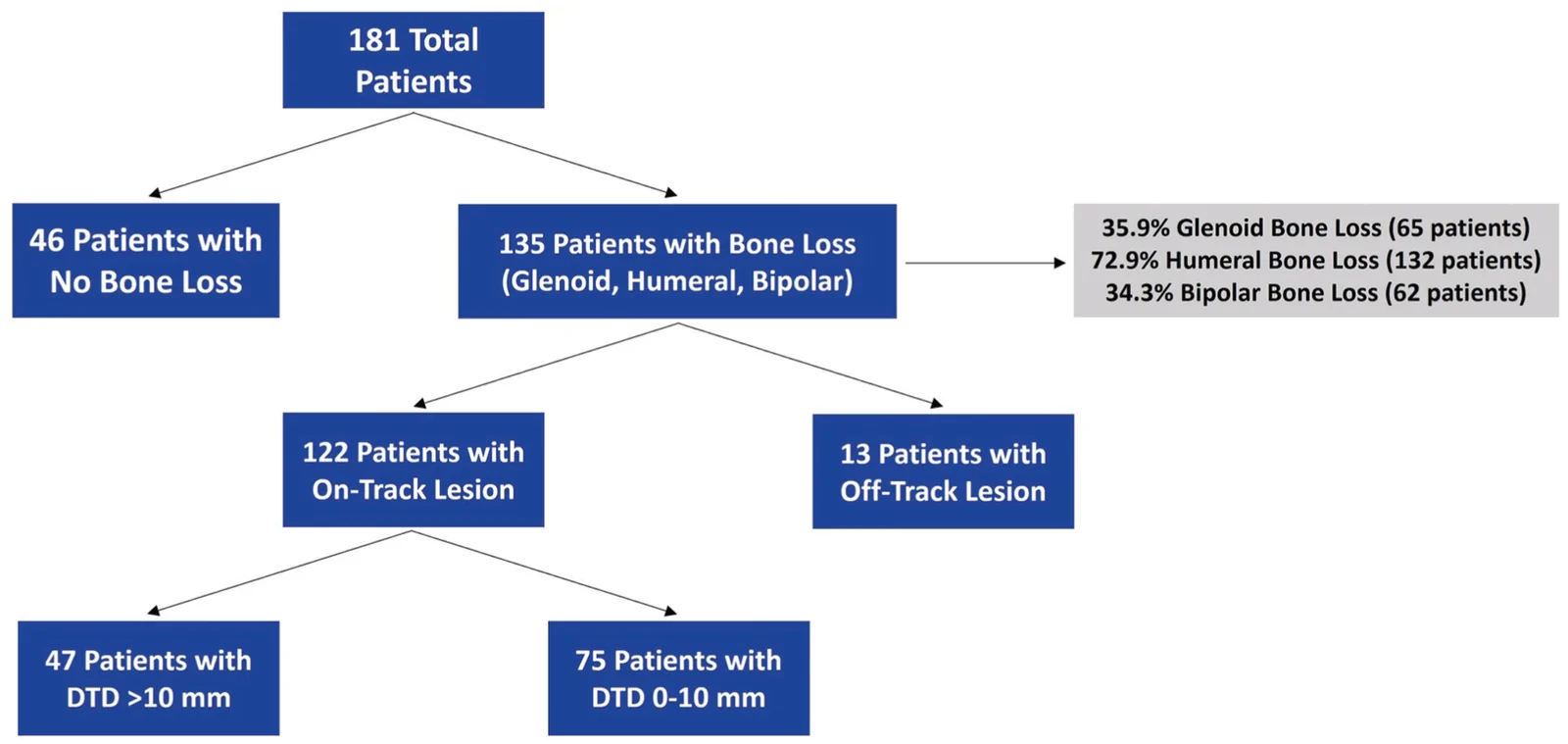
Patient breakdown by bone loss, lesion track, and distance to dislocation (DTD).

### Imaging

Glenoid and humeral bone loss were measured on either standard or arthrogram magnetic resonance imaging (MRI). Glenoid bone loss was measured using the perfect circle method on sagittal T1-weighted MRI sequences.^1,22^ With the sagittal MRI sequence, the most lateral aspect of the glenoid that includes the entirety of the glenoid was used. A circle was then drawn onto the glenoid and the diameter was measured. Bone loss on the glenoid was then measured from the most anterior aspect of the glenoid to the edge of the remaining glenoid and calculated as a percentage of the diameter of the glenoid. Subcritical glenoid bone loss was defined as bone loss >13.5%. The Hill-Sachs interval (HSI) was then recorded by measuring the longest portion of the Hill-Sachs using the axial MRI sequence. The HSI was then measured from the infraspinatus insertion to the most medial aspect of the Hill-Sachs defect.^31^ Utilizing these measurements, we calculated on-track versus off-track lesions and DTD. On-track versus off-track lesions were calculated as follows: Glenoid Track (GT) = [0.83 × Diameter of the Glenoid (D)] − Width of Glenoid Bone Loss (d). Lesions were considered on-track if GT > HSI and off-track if GT < HSI. DTD was then calculated for all on-track lesions (DTD = GT − HSI). All glenoid and humeral bone loss measurements were performed by a senior orthopaedic resident (D.-H.D.); interobserver reliability was not assessed.

### Statistical Analysis

Descriptive analyses were performed to describe the study population. Continuous data are presented as means and standard deviations, while categorical responses are presented as counts and percentages. The chi-square test of independence, Fisher exact test (when appropriate), and Mann-Whitney *U* test were utilized to determine the relationship between bipolar bone loss and descriptive characteristics, previous injury history, and sports and injury characteristics. Univariate and multivariate regression analyses were performed for the entire cohort and sport-related injuries to determine significant risk factors of bipolar bone loss for both samples. Statistical significance was defined using a threshold of *P* < .05. Analyses were performed using IBM SPSS Statistics Version 29.0.1.1 (IBM).

## Results

A total of 181 patients, 79% (n = 143) male, with a mean age of 15.8 ± 1.6 years were available for study. Of these patients, 74.6% (n = 135) presented with bone loss: 35.9% (n = 65) with glenoid bone loss, 72.9% (n = 132) with humeral bone loss, and 34.3% (n = 62) with bipolar bone loss. Overall, 17.7% (n = 32) of patients had subcritical glenoid bone loss, which was defined as ≥13.5%. The majority of patients had on-track lesions (92.8%; n = 168), while 7.2% (n = 13) had off-track lesions and 41.4% (n = 75) had a DTD <10 mm. On initial presentation, 16.5% of patients evaluated at our tertiary referral center had a history of ≥5 dislocations (Table 1). Overall, 78.2% of patients’ injuries occurred while playing a high-impact sport (Figure 2). Surgical stabilization was recommended at the index evaluation in 61.9% (n = 112) of patients.

**Table 1:** Characteristics and Presentation Data of Patients With Recurrent Shoulder Instability*^*a*^*

	Value
Age, y (n = 172)	15.8 (1.6)
BMI, kg/m^2^ (n = 174)	25.4 (5.1)
Sex	
Male	143 (79.0)
Female	38 (21.0)
Race	
Asian	7 (3.9)
Black/AA	46 (25.4)
White	120 (66.3)
>1 race	6 (3.3)
Other	2 (1.1)
Ethnicity	
Hispanic/Latino	42 (23.2)
Non-Hispanic/Latino	139 (76.8)
Injured shoulder	
Right	93 (51.4)
Left	88 (48.6)
Sports injury	
No	31 (17.4)
Yes	147 (82.6)
Sports type	
Baseball	9 (6.1)
Basketball	14 (9.5)
Boxing	3 (2.0)
Bull riding	1 (0.7)
Cheer	2 (1.4)
Football	78 (53.1)
Gymnastics	3 (2.0)
Lacrosse	3 (2.0)
Martial arts	1 (0.7)
Soccer	5 (3.4)
Softball	3 (2.0)
Swimming	4 (2.7)
Tennis	2 (1.4)
Track	1 (0.7)
Volleyball	6 (4.1)
Weightlifting	1 (0.7)
Wrestling	11 (7.5)
Football injury	
No	69 (46.9)
Yes	78 (53.1)
Impact sports type	
Low impact	32 (21.8)
High impact	115 (78.2)
No. of prior dislocations	
0 or 1	123 (72.4)
2-4	19 (11.2)
≥5	28 (16.5)
Glenoid bone loss	
No	116 (64.1)
Yes	65 (35.9)
Subcritical glenoid bone loss	
No	149 (82.3)
Yes	32 (17.7)
Humeral bone loss (HSI)	
No	49 (27.1)
Yes	132 (72.9)
Bipolar bone loss	
No	119 (65.7)
Yes	62 (34.3)
Any bone loss	
No	46 (25.4)
Yes	135 (74.6)
On- vs off-track	
On-track	168 (92.8)
Off-track	13 (7.2)
DTD <10 mm	
No	106 (58.6)
Yes	75 (41.4)

a Data are presented as n (%) or mean (SD). AA, African American; BMI, body mass index; DTD, distance to dislocation; HSI, Hill-Sachs interval.

**Figure 2.**
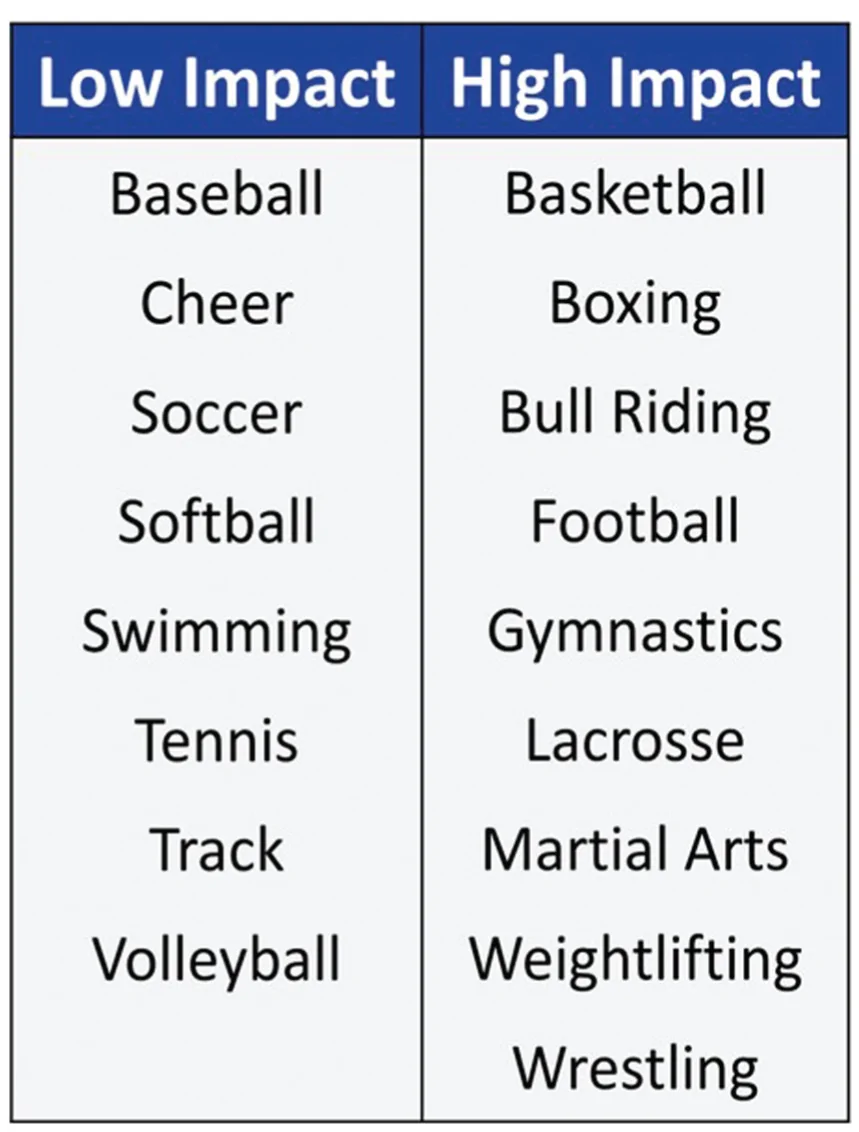
Classification of sports by type of impact.

### First-Time Dislocations

There were 107 of 181 (59%) patients who presented to the clinic after a first-time dislocation. Within this subgroup, the mean age was 15.6 ± 1.6 and 73.8% (n = 79) were male. Overall, 69.2% (n = 74) presented with either glenoid (29.0%; n = 31), humeral (66.4%; n = 71), or bipolar (26.2%; n = 28) bone loss (Table 2). Notably, all 28 cases of bipolar bone loss in this subgroup occurred in male patients. Of these patients, 96.3% (n = 103) had on-track lesions and 3.7% (n = 4) had off-track lesions; 37.4% (n = 40) had a DTD <10 mm after a first-time dislocation. Overall, 12.1% (n = 13) of patients had subcritical glenoid bone loss. Of first-time dislocations, 82.2% (n = 88) were playing sports at the time of injury, with the 4 most common being football (52.3%; n = 46), wrestling (8.0%; n = 7), baseball (6.8%; n = 6), and basketball (6.8%; n = 6).

**Table 2:** Characteristics and Presentation Data of Patients With First-Time Dislocation*^*a*^*

	Value
Age, y (n = 107)	15.6 (1.6)
BMI, kg/m^2^ (n = 101)	25.8 (5.5)
Sex	
Male	79 (73.8)
Female	28 (26.2)
Race	
Asian	5 (4.7)
Black/AA	16 (15.0)
White	82 (76.6)
>1 race	3 (2.8)
Other	1 (0.9)
Ethnicity	
Hispanic/Latino	25 (23.4)
Non-Hispanic/Latino	82 (76.6)
Injured shoulder	
Right	58 (54.2)
Left	49 (45.8)
Sports injury	
No	18 (17.0)
Yes	88 (83.0)
Sports type	
Baseball	6 (6.8)
Basketball	6 (6.8)
Boxing	1 (1.1)
Bull riding	1 (1.1)
Cheer	1 (1.1)
Football	46 (52.3)
Gymnastics	3 (3.4)
Lacrosse	2 (2.3)
Martial arts	1 (1.1)
Softball	2 (2.3)
Swimming	4 (4.5)
Tennis	2 (2.3)
Track	1 (1.1)
Volleyball	5 (5.7)
Wrestling	7 (8.0)
Football injury	
No	42 (47.7)
Yes	46 (52.3)
Impact sports type	
Low impact	21 (23.9)
High impact	67 (76.1)
Glenoid bone loss	
No	76 (71.0)
Yes	31 (29.0)
Subcritical glenoid bone loss	
No	94 (87.9)
Yes	13 (12.1)
Humeral bone loss (HSI)	
No	36 (33.6)
Yes	71 (66.4)
Bipolar bone loss	
No	79 (73.8)
Yes	28 (26.2)
Any bone loss	
No	33 (30.8)
Yes	74 (69.2)
On- vs off-track	
On-track	103 (96.3)
Off-track	4 (3.7)
DTD <10 mm	
No	67 (62.6)
Yes	40 (37.4)

a Data are presented as n (%) or mean (SD). AA, African American; BMI, body mass index; DTD, distance to dislocation; HSI, Hill-Sachs interval.

### Risk Factors for Bipolar Bone Loss

Bipolar bone loss was significantly associated with older age (16.29 vs 15.61 years; *P* = .012), male sex (96.8% vs 69.7%; *P* < .001), sports injury (91.8% vs 77.8%; 2 patients missing in the no bipolar and 1 missing in the bipolar *P* = .019), specifically high-impact sports (94.6% vs 73.6%; *P* = .001), and ≥5 prior dislocations (28.8% vs 9.9%; *P* = .003) (Table 3). A univariate regression analysis looking at these variables determined that increasing age (OR, 1.08; *P* = .009), male sex (OR, 13.01; *P* < .001), sustaining the dislocation while playing sports (OR, 3.20; *P* = .024), and having ≥5 prior dislocations (OR, 4.05; *P* = .001) were significant predictors of bipolar bone loss. When included in a multivariate regression analysis, with BMI included as a control, only male sex (OR, 14.20; *P* = .011) and ≥5 prior dislocations (OR, 3.22; *P* = .026) were identified as predictors of bipolar bone loss (Table 4).

**Table 3:** Characteristics and Presentation Data of Patients With Recurrent Shoulder Instability With Statistical Comparison of Bipolar Bone Loss to No Bipolar Bone Loss*^*a*^*

	No Bipolar Bone Loss (n = 119)	Bipolar Bone Loss (n = 62)	*P* Value
Age, yrs	15.6 ± 1.7	16.3 ± 1.3	.012
Height, cm	171.7 ± 10.6	175.8 ± 7.1	.018
Weight, kg	76.0 ± 19.0	77.2 ± 16.4	.521
BMI, kg/m^2^	25.6 ± 5.0	25.0 ± 5.3	.250
Male sex	83 (69.7)	60 (96.8)	<.001
Right-sided injury	59 (49.6)	34 (54.8)	.502
High-impact sport	76 (73.6)	53 (94.6)	.001
Sports injury	91 (77.8)	56 (91.8)	.019
Football injury	40 (44.0)	38 (67.9)	.005
First-time dislocation	79 (66.9)	28 (45.9)	.006
No. of prior dislocations			.003
0 or 1	89 (80.2)	34 (57.6)	
2-4	11 (9.9)	8 (13.6)	
≥5	11 (9.9)	17 (28.8)	

a Data are presented as n (%) or mean (SD). BMI, body mass index.

**Table 4:** Regression Analysis Evaluating Risk Factors for Bipolar Bone Loss*^*a*^*

	Univariate Analysis			Multivariate Analysis		
	OR	95% CI	*P* Value	OR	95% CI	*P* Value
Age	1.36	1.08-1.71	.009	1.25	0.96-1.63	.100
BMI	0.98	0.92-1.04	.464	0.99	0.92-1.06	.703
Sex						
Male	13.01	3.02-56.15	<.001	14.20	1.82-111.16	.011
Female	Ref			Ref		
Sports injury						
No	Ref			Ref		
Yes	3.20	1.16-8.82	.024	1.79	0.49-6.52	.377
No. of prior dislocations						
0 or 1	Ref			Ref		
2-4	1.90	0.71-5.14	.204	1.83	0.62-5.42	.275
≥5	4.05	1.72-9.51	.001	3.22	1.15-9.00	.026

a BMI, body mass index; OR, odds ratio; Ref, reference.

## Discussion

Adolescent athletes evaluated by us with a history of anterior shoulder instability had a high prevalence of bipolar bone loss on initial presentation. Clinicians should be aware of the high rate of bone loss in this patient population as bone loss has been shown to be an independent risk factor for recurrent shoulder instability. This paper demonstrates that 1 in 3 patients had bipolar bone loss on initial evaluation, with male sex (OR, 14.20; *P* = .011) and the occurrence of ≥5 prior dislocations (OR, 3.22; *P* = .026) as noted correlates. Additionally, within this patient population, 41.4% of patients had a DTD <10 mm. In support of our current understanding of the high rate of recurrence in this population, the increased risk of failure of arthroscopic stabilization may be from a higher percentage of bone loss that has not been reported in this population.

Of clinical significance is the high prevalence of bone loss among those with first-time dislocation in this cohort. Among the 107 (59%) patients evaluated after a single dislocation event, 69.2% were found to have glenoid, humeral, or bipolar bone loss on MRI. Additionally, 37.4% had a DTD <10 mm, a threshold associated with exponentially increased failure after isolated Bankart repair. Interestingly, all 28 cases of patients with bipolar bone loss in the first-time dislocation subgroup occurred in male patients. These findings reinforce the understanding that bone loss is not necessarily a consequence of recurrent instability in adolescents and can be seen even in the index instability event, underscoring the importance of obtaining advanced imaging at first presentation.^6^ Early identification of bone loss in adolescent patients, who already have a high rate of recurrence, is important in the consideration of additional procedures such as remplissage or bone block augmentation when needed.

Prior literature evaluating adolescents with bone loss has focused mainly on glenoid bone loss. Ellis et al^9^ previously reported on bone loss in this patient population and found that 48.2% of adolescents with recurrent traumatic anterior instability had glenoid bone loss, with 27% having critical bone loss defined as >20%. In that study, almost half of the patients with bone loss on advanced imaging did not have radiographic findings of bone loss, suggesting that plain radiographs may underestimate bone loss without advanced imaging. In the current study, inclusive of first-time dislocations and recurrent instability, 35% of the patient population demonstrated glenoid and/or humeral bone loss on advanced imaging.

In regard to prevalence of Hill-Sachs lesions in the adolescent patient population, a recent study that evaluated clinical and radiographic predictors for revision and recurrent instability in adolescent patients found that patients with a higher HSI were more likely to undergo revision surgery than those with a smaller HSI.^8^ Among patients in this group, 64% were found to have evidence of a Hill-Sachs lesion. This is consistent with our findings in that 73% of all patients in our population had the presence of a Hill-Sachs lesion.

The DTD measurement was initially introduced by Barrow et al^2^ and was created to provide more nuance to the glenoid track concept. The authors found that a DTD threshold of ≤10 mm was an exponential risk factor for recurrence with isolated arthroscopic Bankart repair. In our study, 75 (41.4%) patients had a DTD of 0 to 10 mm and 40 (37.4%) patients with a first-time instability event had a DTD of 0 to 10 mm. This finding is significant in that adolescent patients may have a higher prevalence of clinically significant bone loss than previously reported, and this level of bone loss may be one factor leading to a higher failure rate of isolated arthroscopic Bankart repair in this population.

Given the high recurrence rate of anterior shoulder instability, a recent landmark study examined how remplissage affects recurrence rates in patients with anterior shoulder instability through a randomized controlled trial.^30^ The authors found that adding remplissage in cases of subcritical bone loss reduced recurrence rates from 30% to 10% after stabilization, underscoring that appropriately addressing both glenoid- and humeral-sided deficits can significantly reduce recurrence at midterm follow-up. Indications for the addition of remplissage have rapidly changed over the last decade after the introduction of the on-track/off-track concept, with expanding use in off-track and near-track lesions.^5,14^ Remplissage is attractive because of its relatively low complication profile and minimal impact on range of motion, with recent systematic reviews demonstrating no significant difference in external rotation loss compared to isolated Bankart repair or Latarjet procedures.^11,23^ Additionally, remplissage maintains low recurrent instability rates of approximately 10% at midterm follow-up without reducing shoulder range of motion.^19^ For patients with critical glenoid bone loss (>20%) or high-grade subcritical bone loss (>13.5%), open bone block procedures such as the Latarjet technique remain the gold standard, offering superior glenoid arc restoration and low primary redislocation rates of approximately 2.8% to 4.8%.^15^ However, the Latarjet procedure carries higher overall complication rates of 15% to 16%, including hardware complications, graft nonunion or malposition, and nerve injury (1.9%).^4,7^ The most commonly reported nerve injuries involve the axillary, musculocutaneous, and suprascapular nerves, although most cases resolve with nonoperative management.^4^ Given that 37.4% of first-time instability cases in this adolescent cohort demonstrated clinically significant DTD <10 mm, clinicians should carefully evaluate demographic and radiographic risk factors when determining whether isolated Bankart repair is sufficient or whether augmentation with remplissage or bone block procedures is warranted.

In addition to the radiographic measurements that may predispose patients to bipolar bone loss, demographic risk factors found in this study mirror those in other previous studies that have shown risk factors for recurrent instability as well as bone loss.^3,25,28,29,32^ Risk factors for recurrence include older age, male sex, ≥5 dislocations, and high-impact sports participation. Lau et al^16^ compared bipolar bone loss of adolescent versus adult patients with anterior shoulder instability and found similar findings in that there is a greater likelihood of off-track lesions in adolescent patients, and multiple dislocations is an independent risk factor for an off-track lesion. These findings carry important implications, as clinically significant bone loss is common in the adolescent instability population yet frequently underrepresented on standard radiographs. Clinicians should therefore have a low threshold for advanced imaging at initial presentation, as early identification of bone loss allows for more accurate counseling regarding recurrence risk and guides both nonoperative and operative decision-making. Specifically, surgeons should recognize that close to half of this population may have bone loss that may result in a higher risk of failure if not addressed, as multiple studies now support that an isolated arthroscopic Bankart repair is often insufficient in this population.^20,24,26^ Surgical stabilization was recommended at the index evaluation in 61.9% of patients in this cohort. Patients in this cohort were initially evaluated by a range of providers, and some patients who were not offered stabilization at the index visit may have been referred for surgical consultation and offered operative treatment subsequently. The proportion reported here therefore likely underestimates the number of patients ultimately offered surgical stabilization. Our current practice philosophy is to offer early surgical stabilization after an initial shoulder instability event, and this recommendation for surgery likely does not reflect the current amount of patients recommended surgery on presentation after an instability event.

During our initial review of patients evaluated with anterior shoulder instability, 101 of 331 (30.5%) did not have advanced imaging available. These patients were initially evaluated by a range of providers, including advanced practice providers, nonoperative sports medicine specialists, and operative sports medicine surgeons. These findings indicate that a substantial proportion of patients were managed at initial presentation without advanced imaging. To our knowledge, no prior study has reported how often advanced imaging is obtained at initial evaluation in this population. However, the high prevalence of bone loss in our cohort underscores the limitations of relying on plain radiographs alone. Prior studies have shown that standard radiographs frequently fail to identify bone loss, supporting the routine use of MRI at initial evaluation in this age group.

### Limitations

The strengths of this study include its large cohort of adolescent patients and the use of advanced imaging to evaluate bone loss in a high-risk group that has been underreported. The main limitations include its retrospective study design with the potential for selection bias. As a tertiary referral center, our institution may have had a higher percentage of patients with bone loss than seen in the community, which may limit generalizability. Glenoid and humeral bone loss were assessed on T1-weighted sagittal and axial MRI sequences. While computed tomography (CT) has been shown to yield larger bone loss values than MRI in some studies, prior work has also demonstrated that MRI using the best-fit circle technique is accurate and reliable for quantifying glenoid bone loss when compared to CT and arthroscopic measures.^18,27,32^ Furthermore, MRI allows simultaneous evaluation of soft tissue pathology without the radiation exposure seen with CT, which is an important consideration during the evaluation of patients with shoulder instability. Additionally, this study evaluated only patients who underwent advanced MRI. Adolescents with less severe initial injury may not have been referred for advanced imaging and therefore not included in this cohort. This selection bias may have resulted in a higher prevalence of bone loss than would be seen otherwise. All measurements were performed by a single observer, and inter- and intraobserver variability analyses were not performed, which may limit assessment of measurement reproducibility. Although the proportion of patients for whom surgical stabilization was recommended was captured, whether patients ultimately underwent surgery and the specific procedure performed were not recorded, and these outcomes remain an important direction for future work.

## Conclusion

The adolescents in our series with anterior shoulder instability demonstrated a high prevalence of bone loss, with 75% presenting with glenoid, humeral, or bipolar bone loss. Among the entire cohort, 34.3% had bipolar bone loss, and 41.4% of all patients had on-track lesions with a DTD <10 mm. Additionally, 7.2% of patients with bone loss presented with off-track lesions and 17.7% with subcritical glenoid bone loss. Among those with first-time dislocation, 69.2% presented with bone loss and 37.4% had DTD <10 mm. Risk factors independently associated with bipolar bone loss included male sex and ≥5 prior dislocations. These findings underscore the high prevalence of clinically significant bone loss in this population and suggest that isolated arthroscopic Bankart reconstruction may be insufficient for many adolescent patients. Treatment teams should consider adjunct procedures such as remplissage, open Bankart, or bone block augmentation, particularly in patients with identified risk factors.
